# Next-Generation Probiotics in Poultry: From Conventional Strains to Engineered and Synbiotic Approaches

**DOI:** 10.3390/microorganisms14081680

**Published:** 2026-07-31

**Authors:** Tahani Al-Surrayai, Sheikh Shreaz, Hanan Al-Khalaifah

**Affiliations:** Food Security Program, Environment and Life Sciences Research Center, Kuwait Institute for Scientific Research, P.O. Box 24885, Kuwait City 13109, Kuwait; tsuraia@kisr.edu.kw

**Keywords:** next-generation probiotics, poultry production, gut microbiome, antimicrobial resistance, precision nutrition

## Abstract

The global poultry industry faces a major transition driven by bans on antibiotic growth promoters (AGPs) and rising antimicrobial resistance threats. This has intensified the search for effective, sustainable alternatives to maintain productivity, animal health, and food safety. This review provides a comprehensive overview of next-generation probiotics (NGPs) and their evolving role in poultry production, highlighting their mechanisms of action, technological advancements, and applications for improving performance and health. Recent studies were systematically reviewed, covering conventional and NGPs, multi-omics-driven strain identification strategies, engineered microbial systems, and advanced delivery technologies. Key functional aspects, including gut microbiota modulation, immune responses, pathogen control, and productivity metrics were thoroughly assessed. NGPs exhibit enhanced capabilities over traditional probiotics, including improved gut colonization, targeted antimicrobial activity, and precise host immune modulation. Advances in synthetic biology, microencapsulation, and synbiotic formulations enhance their efficacy and stability. Additionally, NGPs also demonstrate significant potential in improving growth performance, feed conversion ratios, intestinal integrity, and enteric pathogen resistance. However, challenges such as strain-specific variability, regulatory constraints, and limited field-level validation persist. NGPs offer a promising and sustainable strategy for modern poultry production. Future integration with precision microbiome engineering, artificial intelligence, and sustainable farming will be essential to realize their full potential and ensure widespread adoption.

## 1. Introduction

Poultry production ranks among the rapidly developing industries in global agriculture, playing a critical role in food and nutritional security owing to cost-effectiveness and operational efficiency. The rising demand of high-quality animal protein, driven by growth of population and urbanization, has placed considerable pressure on poultry production systems throughout the world [1]. Compared to other livestock sectors, poultry production is characterized by rapid growth, superior feed conversion efficiency, and high yield of both meat and eggs [2]. In recent years, the poultry industry has undergone significant transformation, incorporating advances in nutrition, genetics, and management to enhance both productivity and sustainability [3]. Beyond productivity, the poultry industry makes a substantial contribution to rural livelihoods and economic development, particularly in developing nations [4]. As growing environmental and sustainability concerns reshape food production, alternative approaches to boosting system productivity are gaining traction as ecological balanced solutions, with probiotics and prebiotics figuring among the most promising ones [5].

Antibiotic growth promoters (AGPs) were widely used in poultry feed for decades to accelerate growth, enhance feed efficiency, and prevent disease outbreaks. However, their indiscriminate and uncontrolled use has been strongly associated with the emergence and spread of antimicrobial-resistant bacteria, posing a serious threat to both animals and human health [6]. In response, numerous countries have adopted strict regulations or outright bans on AGP use in animal production [7]. The withdrawal of AGPs has created significant problems for the poultry industry, including increased susceptibility to enteric diseases, decreased growth performance, and economic losses [2]. Therefore, there is an urgent need to identify safe and effective alternatives that can maintain productivity without contributing to antimicrobial resistance (AMR) [8]. Probiotics and advanced microbial interventions have emerged as promising candidates in this regard, offering tangible benefits for gut health and pathogen suppression [5,9].

Gut health has emerged as a central determinant of poultry performance, governing nutrient digestion, immune response, and overall physiological function. The gastrointestinal microbial community in poultry constitutes a highly complex and dynamic ecosystem that plays a crucial role in maintaining intestinal homeostasis and resisting pathogen colonization [10]. A well-balanced gut microbiota enhances nutrient absorption and increased growth performance and feed efficiency [11]. Disruption to gut microbial homeostasis, whether triggered by stress, pathogens, or dietary alterations, can compromise intestinal integrity, leading to reduced productivity and increased disease susceptibility [12]. Additionally, the interaction between gut microbiota and the host immune system plays a key role in controlling inflammatory reactions and overall health conditions [13]. Thus, modulating the gut microbiome through probiotic supplementation has gained increasing traction as a strategy for optimizing poultry performance [14]. While conventional probiotics have been shown to produce positive consequences in poultry, their outcomes often remain inconsistent due to strain-specific variability, environmental influences, and limited understanding of host–microbe interactions. Conventional probiotic strains such as *Lactobacillus* and *Bacillus* may be ill-equipped to address the complex challenges of intensive modern poultry production systems [15]. The investigation of next-generation probiotics (NGPs) has occurred as a reflection of even more advanced research on the microbiome. NGPs can be defined as highly specialized, host-derived microbial strains identified through precision-based technologies to target and regulate specific physiological functions, such as immune modulation and pathogen resistance [16,17]. Consequently, NGPs represent a viable future for the field of poultry nutrition and health management, which complies with the objectives of sustainable and antibiotic-free production [15].

**Figure 1:**
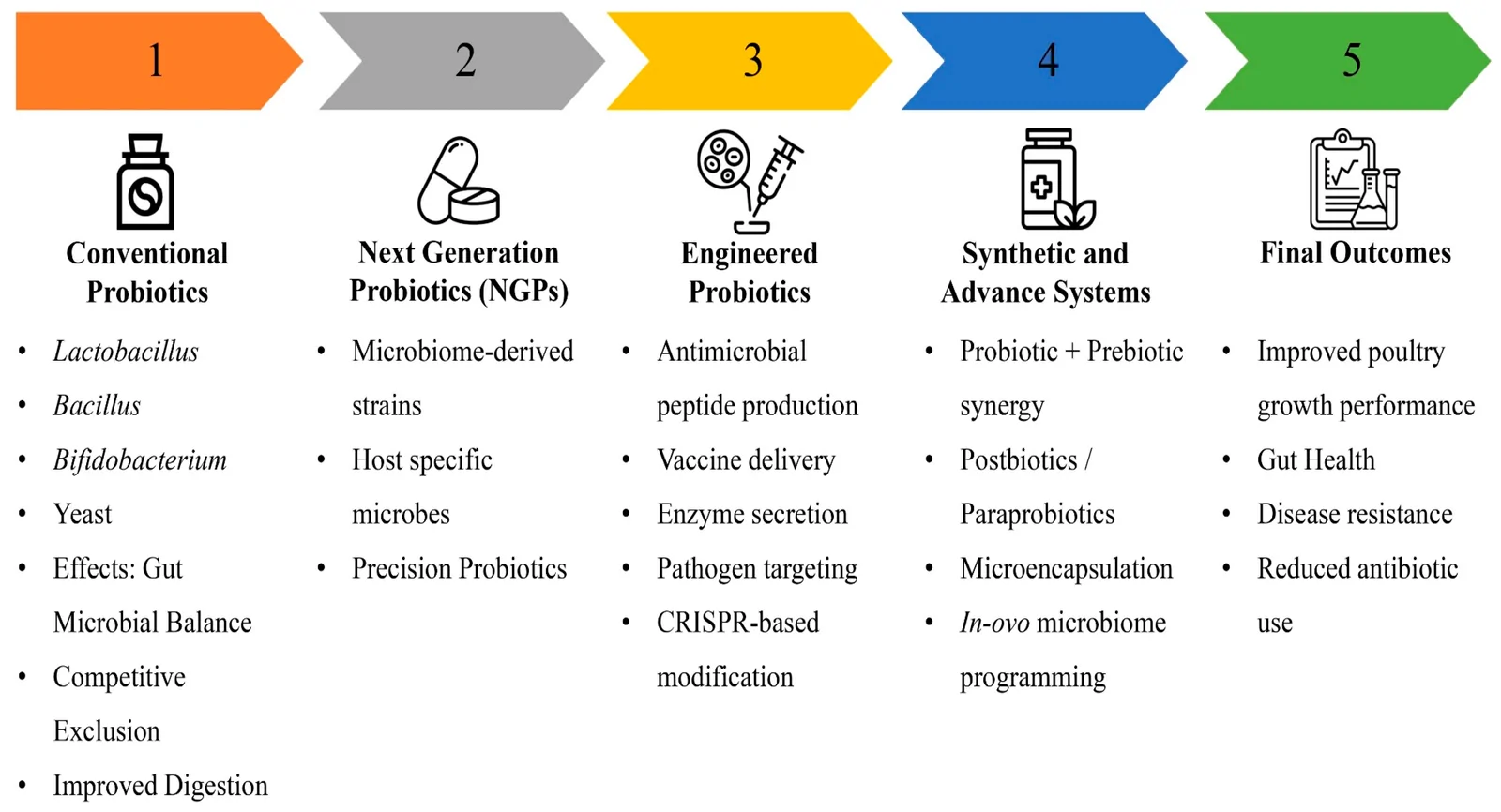
Evolution of probiotic strategies in poultry: transition from conventional strains to next-generation, engineered, and advanced synbiotic systems for precision microbiome modulation [15,16,18,19].

The evolution of probiotic strategies in poultry production reflects a fundamental shift from traditional microorganism supplementation towards innovative, precision-driven approaches. This transformation encompasses the development of NGPs, engineered microbial systems, and synbiotic formulations designed to improve functional efficacy, target specificity, and long-term sustainability. A comprehensive overview of this progression, highlighting the key stages and corresponding functional outcomes in poultry production, is illustrated in Figure 1 [15,16,18,19].

As demonstrated in Figure 1, probiotic development has progressed well beyond the use of traditional strains such as *Lactobacillus*, *Bacillus*, and *Bifidobacterium* toward more advanced methods, including microbiome-derived NGPs and genetically engineered strains with defined functional activities. Unlike probiotics, which rely on the successful colonization of live microorganisms, postbiotics consist of non-viable microbial metabolites and components that provide health benefits to the host without the challenges of maintaining bacterial viability. Collectively, these innovations translate into improved growth performance, better gut health, enhanced disease resistance, and a reduction in antibiotic dependence within poultry production systems [15,16,19].

In keeping with this evolutionary view, this review provides an in-depth overview of both traditional and forthcoming probiotics, their activity, technology, and application in contemporary poultry production. The purpose of this review is to present a summative understanding of the changing role of probiotics in poultry production, in particular next-generation approaches. This encompasses traditional probiotic interventions to novel multifaceted innovations, including engineered probiotics, synbiotic, and precision microbial therapeutics. Additionally, this review will document the mechanisms of action, delivery systems, and practical implications of these emerging technologies, along with remarks on current challenges and a future research agenda in the field of poultry health and nutrition.

## 2. Conventional Probiotics in Poultry: Current Status and Limitations

Functional feed additives such as conventional probiotics have been widely used in poultry production to promote gut health, nutrient utilization, and overall productivity in antibiotic-free production systems. Most of these probiotics are established microbial genera (such as lactic acid bacteria, *Bacillus*, *Bifidobacterium*, and yeast genera) and have been shown to have positive effects on host physiology and intestinal microbial balance [2,20,21]. They have a variety of mechanisms that support their application, including competitive exclusion of pathogens, antimicrobial metabolite production, improvement of intestinal integrity, and regulation of immune responses [5,22,23,24].

A number of reports have highlighted strain-specific differences in probiotic effect, and their effects have been shown to enhance the growth performance, feed efficiency, immune responses, and gut microbiota balance in poultry systems [14,25,26,27]. Table 1 displays a consolidated description of the most widely used probiotic strains, and their origins, mechanisms, and functional advantages in poultry production [2,5,12,14,27,28,29,30,31,32,33,34,35,36,37].

The strains summarized in Table 1 highlight the diversity of conventional probiotics and their multifunctional roles in poultry systems. Despite their established benefits, the effectiveness of these probiotics can vary depending on strain specificity, environmental conditions, and host-related factors, which are further discussed in the following sections.

### 2.1. Commonly Used Probiotic Strains

The bacterial genera most commonly used as conventional probiotics in poultry production, which have a long-established positive impact on host physiology and gut microbiota balance, are *Lactobacillus*, *Bacillus*, *Bifidobacterium*, and yeast species [20]. The choice of these microorganisms is determined by their capacity to survive gastrointestinal environments and deliver positive functional effects on the host [21]. Among them, members of the *Lactobacillaceae* family are widely used because of their ability to generate lactic acid to stabilize the pH, thereby preventing pathogenic bacteria and stabilizing the gut [2]. Recent studies have highlighted the use of lactic acid bacteria-based probiotic formulations in poultry; these studies confirm the beneficial effect of these formulations on gut and overall poultry health [38]. Likewise, *Bacillus* species, especially spore-forming species, are preferred due to their high stability during feed processing and their enzyme production, which facilitates nutrient digestion [34].

Moreover, the *Bifidobacterium* species enhance gut health by producing (SCFAs) and regulating the intestinal microbial community [12]. Yeast-based probiotics, including *Saccharomyces cerevisiae* and *Saccharomyces boulardii*, have attracted considerable attention in poultry production. For example, their practical application in poultry diets is widely used to bind enteric pathogens, mitigate the adverse effects of mycotoxins in contaminated feed, and enhance nutrient digestibility. Furthermore, the dietary inclusion of these specific yeast strains has been shown to effectively activate the host’s innate immunity and improve overall flock performance [27,36,37]. Although popular, the efficacy of these traditional probiotic strains can vary, depending on the strain specificity, environmental factors, and host factors, underscoring the need for more specific and precise microbial interventions [15].

### 2.2. Mechanisms of Action in the Avian Gut

Probiotics exert many interconnected effects on the gut microbiota, intestinal integrity, and host immune responses. Advantageous microbes enhance intestinal health through a multifaceted approach. Primarily, they utilize competitive exclusion to occupy ecological niches and prevent pathogenic colonization [22]. Beyond simply displacing pathogens, these beneficial microbes actively reorganize the host’s intestinal architecture. By stimulating villi growth and upregulating the expression of tight junction proteins, they effectively reduce intestinal permeability and fortify the epithelial barrier, ultimately optimizing nutrient absorption [23,39]. In our view, this dual action, simultaneously defending against pathogens while structurally fortifying the gut, highlights why probiotics remain a cornerstone of modern poultry management. Similarly, it has been showed that probiotic supplementation combined with fermented feed components enhances intestinal barrier-related gene expression in broiler chickens [40]. Another complex process is the host immune system modulation, where probiotics regulate cytokine production and potentiate both innate and adaptive immune responses, improving disease resistance in poultry [41]. Moreover, probiotics produce antimicrobial metabolites that inhibit harmful microorganisms and promote beneficial ones [5]. Taken together, these processes underscore the complex role of probiotics in maintaining gut homeostasis and improving the overall health and performance of poultry [42]. Furthermore, while the production of SCFAs is frequently cited as a primary mechanism of probiotic efficacy, this concept requires careful ecological contextualization. Approximately 98% of all gut anaerobes, including known enteric pathogens such as *Clostridium perfringens*, *Escherichia coli*, and *Salmonella*, are baseline SCFA producers. Because the chicken caecum already harbors hundreds of indigenous SCFA-producing species, the introduction of a single probiotic strain does not meaningfully increase the absolute bulk SCFA pool. Rather than simple SCFA addition, the true mechanistic value of advanced probiotic interventions lies in their ability to drive targeted shifts in the SCFA profile (such as optimizing butyrate-to-acetate ratios) and facilitating complex cross-feeding networks that selectively stimulate indigenous beneficial taxa over pathogenic ones [43,44,45].

### 2.3. Effects on Growth, Health, and Productivity

Dietary probiotics in poultry diets have been consistently linked to enhanced growth, feed efficiency, and productivity. Such advantages are mostly related to the improved digestion and absorption, resulting in better feed-to-weight ratios and higher weight gain [26]. Probiotics have also enhanced immune response and disease resistance, reducing disease outbreaks in poultry flocks [25]. By crucially modulating the gut microbiota, they help to maintain a balanced microbial ecosystem, which is essential for optimal health and productivity [14]. Besides growth and immunity, probiotics improve physiological parameters such as stress tolerance and metabolic efficiency, thereby enhancing bird performance across diverse production environments [46]. In addition, certain probiotic strains, particularly spore-forming bacteria, have demonstrated substantial increases in growth rates and immune responses due to their stability and functional viability in the gastrointestinal tract [47]. Collectively, these findings highlight the significance of probiotics as functional feed additives that support enhanced productivity in antibiotic-free poultry production systems [48].

### 2.4. Strain-Specific Variability and Inconsistent Efficacy

Although probiotic poultry production benefits are well-documented, several constraints and gaps in knowledge still limit their widespread adoption. Key effectiveness varies considerably depending on the strain, dose, and environmental and host-specific factors [49]. The absence of standardized protocols for probiotic selection, formulation, and administration leads to uneven outcomes across studies and production systems [50]. Given these pervasive inconsistencies, a provocative hypothesis must be considered: rather than deterministic mechanistic differences, could the variable efficacy of conventional probiotics sometimes merely reflect random statistical variations within highly complex biological systems? Challenging the traditional paradigm by acknowledging this potential for randomness forces a critical re-evaluation of experimental designs and helps to avoid passive confirmation bias in future microbiome research [51,52]. Moreover, limited field-level validation and scalability pose additional obstacles to their commercial adoption in poultry production [53]. Deeper insights into host–microbiome interactions and probiotic mechanism are needed, as current knowledge remains insufficient to fully optimize probiotics [49]. Moreover, regulatory challenges, including approval, safety assessment, and the potential transfer of antimicrobial resistance genes, must also be addressed [50]. Overcoming these limitations requires a rigorous research approach and enhanced standardization to unlock the full benefits of probiotics in modern poultry production systems [53].

## 3. Next-Generation Probiotics

**Figure 2:**
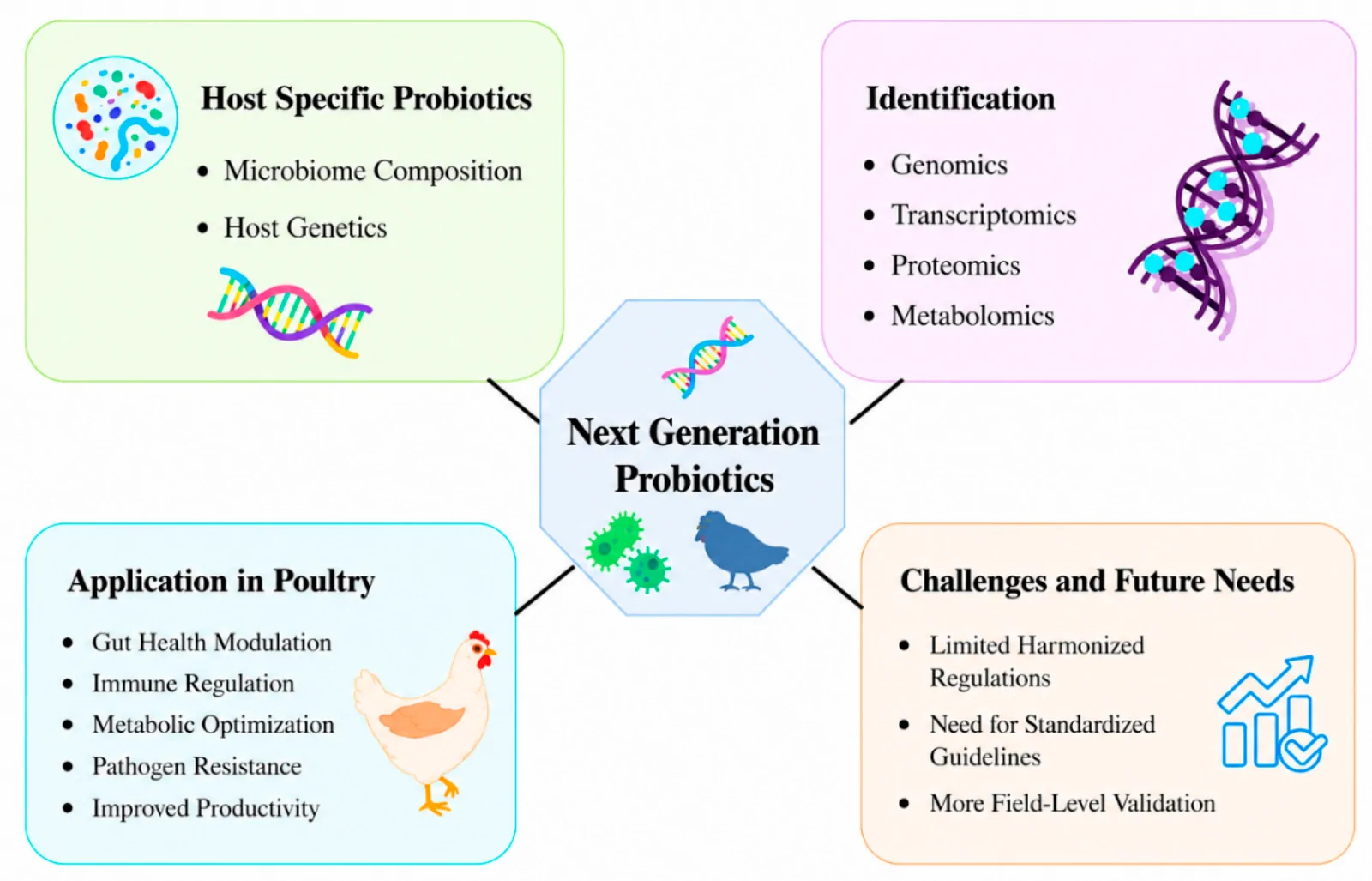
Integrated conceptual framework of NGPs depicting their precision-based identification, functional mechanisms, and applications in sustainable poultry production [54,55,56,57,58].

NGPs represent a significant advance in poultry health and productivity for antibiotic-free production. Unlike traditional probiotics, NGPs leverage modern microbiome studies and precision-based methods to target specific physiological functions within the host. They integrate cutting-edge technologies, including multi-omics, synthetic biology, and microbiome engineering, to increase their functional efficacy and specificity. The conceptual representation of NGPs’ key elements, processes, and application in poultry systems is presented in Figure 2 [54,55,56,57,58].

The framework in Figure 2 summarizes the transition from conventional probiotic approaches to advanced, precision-driven microbial interventions. It integrates the core elements that define NGPs, including their scientific foundation, functional attributes, and practical implications for poultry production systems. These aspects are discussed in detail in the following subsections.

### 3.1. Definition and Concept of NGPs

NGPs represent an advanced category of microbial-based interventions that has gone beyond the traditional definitions of probiotics through their precision, enhanced functionality, and therapeutic potential. Unlike traditional probiotics, which are often selected based on historical use and general benefits, NGPs are characterized by scientific evidence demonstrating their targeted effects on host health [59]. These emerging microbial candidates are increasing considered as a biotherapeutic agent capable of addressing complex health issues in both humans and animals [54]. At their core, NGPs reflect the shift from generalized gut health promoters toward highly specialized microbial agents with defined mechanisms of action. They are typically sourced from the host microbiome and selected based on their capacity to regulate key physiological functions, including immune function, metabolism, and pathogen resistance [17]. While established conventional strains like *Lactiplantibacillus plantarum* offer known baseline benefits [60], strictly defined NGPs expand upon these foundational effects by utilizing novel, host-derived anaerobes to promote targeted immunomodulation, gut microbiota modification, and increased production efficiency. However, to truly leverage the avian microbiome, researchers are increasingly looking beyond conventional lactic acid bacteria. The chicken gastrointestinal tract harbors roughly 1000 distinct bacterial species, yet historical interventions have narrowly focused on a handful of genera. Next-generation approaches emphasize the characterization of core, host-derived strict anaerobes, such as *Blautia*, *Bacteroides*, *Faecalibacterium*, *Butyricicoccus*, and *Sutterella*, which possess highly specific metabolic pathways (such as targeted SCFA profiles) that conventional strains lack [61]. This transition is particularly important in poultry production, driven by the rising demand for antibiotic-free systems and sustainable disease management strategies [57].

Moreover, NGPs address the shortcomings of conventional strains, offering superior efficacy, stability, and specificity even amid varying environmental and physiological conditions [62]. Advances in microbial biotechnology have enabled the development of NGPs that are safer and exhibit superior functional characteristics, rendering them highly suitable for intensive poultry production systems [63].

### 3.2. Identification Through Multi-Omics and Microbiome Analysis

NGPs are identified using high-quality multi-omics methods, such as genomics, transcriptomics, proteomics, and metabolomics. These enable characterization of beneficial microorganisms, their host interactions, and their potential as new probiotic candidates [55]. Multi-omics analyses have substantially advanced our understanding of microbial metabolism and functional abilities, empowering researchers to select strains with targeted health-promoting traits [64]. This integrated approach additionally elucidates the complex host–microbiome interactions essential for designing targeted probiotic interventions [65]. Poultry microbiome studies have yielded critical insights into how microbial communities influence growth performance, immunity, and disease resistance, guiding the selection of NGP strains [63]. These innovations support probiotic development, moving beyond traditional trial-and-error methods [55].

### 3.3. Host-Specific and Precision Probiotics

A key innovation in next-generation microbial interventions is host-specific, precision probiotics, which tailor probiotic strains to specific host species and physiological conditions. Unlike traditional probiotics, which are applied across diverse hosts, precision probiotics are designed based on each host’s microbiome and genetic background [56]. Recent research has demonstrated that some probiotics are host-specific, influencing their gut colonization efficacy and functional performance [66]. This host specificity is particularly relevant in poultry, where microbiome alteration can profoundly affect probiotic efficacy and overall production outcomes [67]. Furthermore, integrating microbiome data with host-specific responses has enabled personalized probiotic therapies [68]. These targeted strategies deliver superior efficacy by aligning probiotic action with host needs, thereby enhancing performance and reducing result variability [57]. Overall, precision probiotics offer tailored solutions to address limitations in broad-spectrum probiotic applications [56].

### 3.4. Safety and Regulatory Considerations

Developing and using NGPs requires stringent safety and regulatory examination to confirm their appropriateness for animal production systems. Assessing possible risks from microbial strains, such as pathogenicity, toxicity, and antimicrobial resistance gene transfer, remains a primary concern [69]. Regulatory frameworks for probiotic usage differ by geographical area, highlighting the need for unified rules to ensure product quality, safety, and effectiveness [70]. During the last few years, an increasing focus has been placed on the development of broad regulatory policies that tackle the intricacies related to emerging probiotic technologies, such as next-generation and engineered strains [58]. Also, safety issues based on dosage, formulation, and long-term effects should be critically considered with the help of well-designed studies before they can be applied on a large scale in poultry production. The absence of internationally recognized standards and standardized testing conditions is one of the major issues in the commercialization of NGPs [71]. Addressing these regulatory and safety issues is essential to build consumer confidence and allow NGPs for use in sustainable poultry production systems [70].

## 4. Engineered Probiotics and Synthetic Biology Approaches

Engineered probiotics represent a paradigm shift in chicken nutrition and health care, as they combine genetic engineering with synthetic biology to improve the functional abilities of valuable microbes. These methods allow for the creation of highly specialized probiotic strains with enhanced stability, precision, and specific biological effect to overcome the weaknesses of conventional probiotics in today’s modern intensive production systems [72,73,74,75]. The use of more sophisticated molecular technologies, such as clustered regularly interspaced short palindromic repeats (CRISPR)-based genome editing and synthetic biology platforms, has accelerated the development of probiotics with advanced antimicrobial, immunomodulatory, and metabolic capabilities [76,77]. Table 2 presents a systematic review of the key engineering strategies, tools, functional applications, and related challenges [8,10,26,70,72,75,77,78,79,80,81,82,83,84,85].

The strategies outlined in Table 2 demonstrate the diverse applications of genetic engineering and synthetic biology in enhancing probiotic functionality for poultry systems. These innovations enable the development of precision-targeted microbial interventions capable of improving gut health, nutrient utilization, immune responses, and disease resistance. However, despite their promising potential, the successful implementation of engineered probiotics requires addressing challenges related to biosafety, regulatory approval, and large-scale validation.

### 4.1. Rationale for Genetic Engineering of Probiotic Strains

Genetic engineering of probiotics is a promising technique for enhancing the ability of these microbes beyond what is naturally achievable. Traditional probiotic strains exhibit variability in their performance under different environmental and host conditions. Therefore, modification is needed to improve their efficacy and stability [86]. Genetic engineering provides an efficient means to enhance probiotic characteristics, such as increased metabolic activity and protection against the adverse effects of the gastrointestinal tract environment [73]. Engineered probiotics can be customized to perform desired biological tasks, such as targeting pathogens and regulating host immune responses. They provide improved efficacy compared to conventional probiotics [74].

Recent improvements in molecular techniques have enabled the development of highly resilient probiotic strains, which can serve as defenders against enteric pathogens in poultry [87]. Moreover, these sophisticated genetic techniques applied to lactic acid bacteria are yielding improved probiotic strains [76].

### 4.2. Engineering Strategies for Functional Enhancement

#### 4.2.1. Enhanced Antimicrobial Peptide Production

Heterologous expression of antimicrobial peptides (AMPs) in engineered probiotics is another promising technique, enabling the effective inhibition of pathogenic microorganisms in the gut. AMPs can be incorporated into engineered probiotic strains to increase their antimicrobial efficiency and spectrum of activities [78]. Introducing genes encoding AMPs enables these probiotics to serve as effective delivery systems, producing AMPs on site in the host gut and thus ensuring effective disease prevention in poultry [79]. This approach not only boosts pathogen elimination but also reduces the poultry production system’s reliance on antibiotic treatment [88]. Engineered probiotics can additionally secrete molecules that help to prevent antibiotics from causing damage to beneficial gut microbes, ensuring effective prevention of antibiotic resistance [89]. Such innovations highlight the potential of engineered probiotics as multifunctional tools in sustainable poultry production systems [73].

#### 4.2.2. Pathogen-Specific Targeting

Engineering of probiotics allows the utilization of highly specific targeting strategies, such as receptor binding and competitive exclusion. They can be modified to specifically recognize pathogens and inhibit their growth in the gastrointestinal tract [87]. This approach enhances selectivity for suppressing pathogens while maintaining beneficial microorganisms [74]. In addition, synthetic biology advances now allow engineering of probiotics capable of detecting pathogens and producing antimicrobial agents against them [76]. This specificity marks a considerable improvement over traditional probiotics, which rely on nonspecific modes of action [90]. The future potential of developing such probiotic technologies is very promising in controlling diseases and reducing pathogens’ load in broiler chickens [86].

#### 4.2.3. Delivery of Vaccines and Immunomodulatory Molecules

Genetically engineered probiotics have attracted interest for delivering vaccines and immunomodulatory compounds to poultry. This approach leverages their capability to directly influence the host’s immune response. They can deliver specific antigens or immunomodulators to promote protective immunity [80]. These probiotics serve as effective vaccine delivery tools, offering improved safety, ease of administration, and mucosal immunity in poultry [81]. In addition, probiotics demonstrate effectiveness in promoting such immune responses through their antioxidant and immunomodulatory properties, enhancing disease resistance [91]. Moreover, incorporating immunomodulators into probiotics boosts their efficiency, as these organisms stimulate targeted immunity. For example, cytokines and toll-like receptor agonists enhance immune responses to antigens [92]. Finally, advancements in probiotics encapsulation improve their stability and efficacy in poultry [93].

#### 4.2.4. Enzyme Secretion for Improved Nutrient Utilization

Probiotics can be engineered to secrete enzymes that promote the breakdown and absorption of feed nutrients in poultry. Such enzymes include phytase, proteases, and carbohydrases, which play an integral role in the breakdown of complex components of feed components, making more nutrients available for uptake [82]. Evidence shows that enzyme-producing probiotics improve the FCR, growth rates, and digestive processes in the gastrointestinal tract [26]. Such probiotics may also influence the composition of the gut microbiota to ensure that nutrients are absorbed optimally and metabolized efficiently [94]. Further, combining exogenous enzymes with probiotics ensures the maintenance of gut health and stability, especially when rearing chickens intensively [95].

### 4.3. CRISPR and Synthetic Biology Tools in Probiotic Development

CRISPR-Cas systems and synthetic biology have greatly transformed probiotic engineering, offering unprecedented efficiency and accuracy in genetic modification. Unlike conventional genetic modification methods, CRISPR provides higher specificity and versatility, making it possible to manipulate genes without causing any unintended modifications or errors [72]. This has led to novel probiotic strains with desired functional properties. CRISPR enables adding or deleting genes to control essential physiological processes, such as synthesizing beneficial compounds, degrading undesirable metabolites, or inhibiting pathogens in the intestines [77]. Thus, CRISPR expands probiotic engineering, creating microbes that surpass natural strains [72].

Besides gene editing, synthetic biology offers a methodology for building genetic circuits, which facilitate the ability of engineered probiotics to sense and react to external stimuli. “Smart probiotics” can detect specific physiological factors, such as pathogens and inflammation mediators, and trigger specific reactions, like producing antimicrobial peptides and immunomodulators [96,97]. These dynamic and adaptive mechanisms have advanced therapeutic probiotics [75]. Moreover, combining CRISPR and synthetic biology yields biohybrid systems, with genetically modified probiotics serving as living vehicles for targeted drug delivery [75,77]. Therapeutically, this ensures efficient drug administration while minimizing host adverse effects, particularly in poultry via localized digestive tract delivery [72].

However, ensuring the stability and survival of CRISPR- and synthetic biology-engineered probiotics is crucial for practical applications [98]. Lyophilization and cryoprotectants preserve their integrity under commercial conditions [84]. Thus, an improved preservation method is essential for scaling these strains in poultry farming [75]. Although significant progress has been made, challenges remain, including regulatory issues, safety concerns, and field validation [77]. The successful resolution of those obstacles will determine whether CRISPR and synthetic biology will gain more traction in improving poultry production and eventually become sustainable practices [96]. In summary, they represent a transformative approach to microbiome engineering.

### 4.4. Biosafety, Ethical, and Regulatory Considerations

Engineered probiotics for poultry rearing raise biosafety, ethical, and regulatory concerns. First, their safety remains a major concern that requires consideration, as they may pose ecological hazards via genetic exchange and horizontal gene transfer (HGT) [85]. Crucially, the prospect of releasing genetically modified organisms (GMOs) into the open environment through poultry shedding poses unpredictable and potentially irreversible ecological risks. Furthermore, the fundamental necessity of engineering conventional strains, such as *Lactobacillus* or *Bacillus*, is highly questionable given the current state of microbiome research. The avian gastrointestinal tract harbors hundreds of uncharacterized, naturally occurring species. Before resorting to the creation and environmental release of synthetic GMOs, the scientific community must prioritize the isolation, characterization, and testing of untapped natural candidates such as *Blautia*, *Desulfovibrio*, *Bacteroides*, *Faecalibacterium*, and *Butyricicoccus*. Leveraging this vast, existing natural biodiversity offers a vastly safer and highly promising pathway for NGPs without the profound environmental risks associated with genetic modification [2,15]. Ethical and legal issues, including intellectual property rights and probiotics commercialization, must also be addressed [99]. Moreover, microbial research and development must comply with existing safety and ethical regulations [100]. In addition, there is a need to develop measures for ensuring the proper use of engineered probiotics in the poultry industry by enforcing proper biosafety and antimicrobial stewardship principles [101]. Technological advances in microbiome and synthetic biology raise further challenges for their application in livestock rearing [102].

## 5. Synbiotics and Emerging Functional Combinations

**Figure 3:**
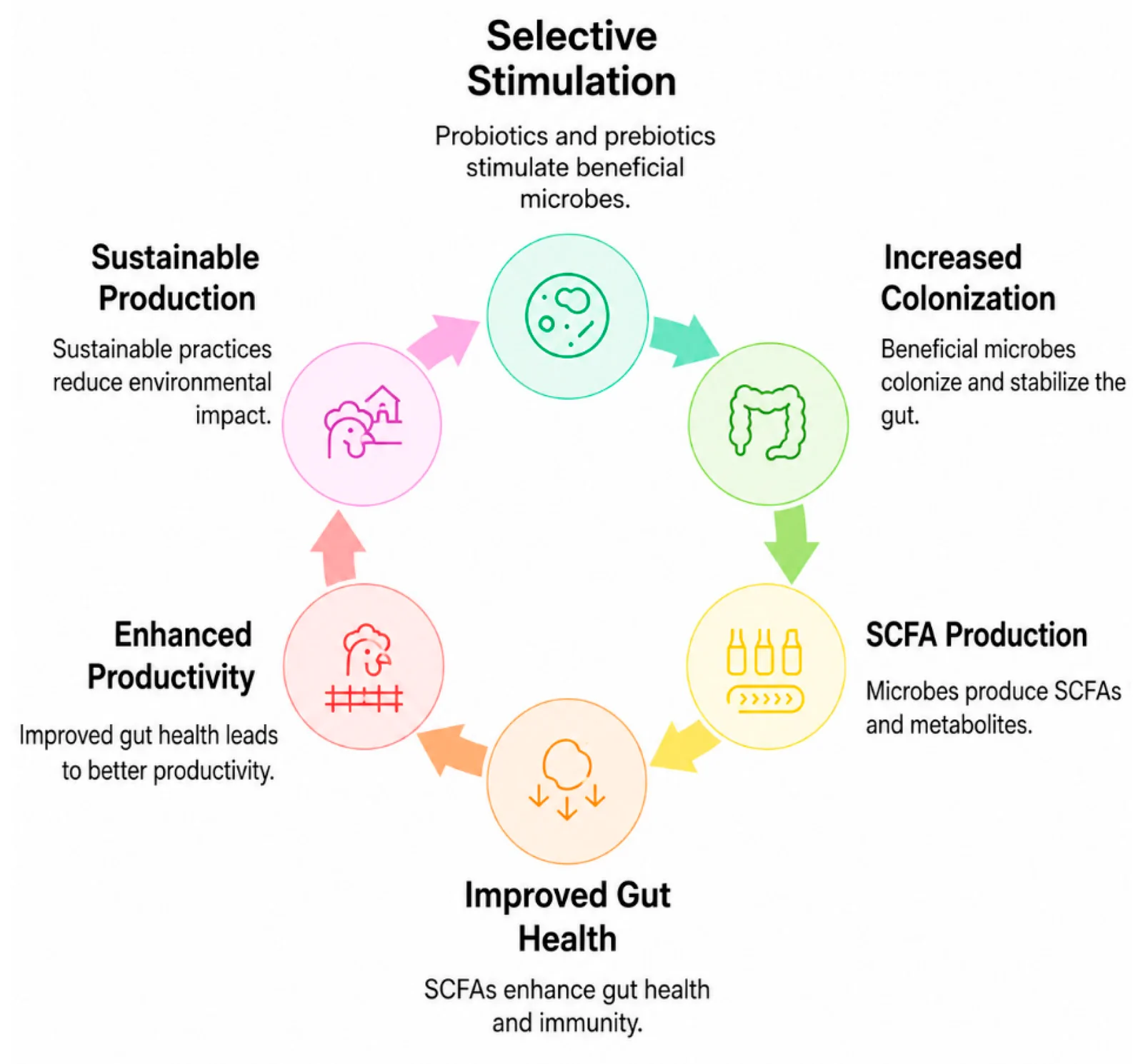
A conceptual illustration of probiotic–prebiotic synergy demonstrating the sequential process of selective microbial stimulation, enhanced colonization of beneficial gut microbiota, increased production of SCFAs, improved gut health and immune function, leading to enhanced productivity and sustainable poultry production systems [18,103,104].

Synbiotics represent a sophisticated nutritional concept involving probiotics combined with prebiotics to optimize intestinal microbiota composition, improve nutrient absorption, and boost immunity in birds. These synergistic interactions promote proliferation and effectiveness of beneficial microflora, facilitating superior intestinal health and increased productivity in poultry farming [103,104,105]. Figure 3 depicts a conceptual model of the synbiotic cycle, illustrating the interaction of its components and their effects in poultry [18,103,104].

### 5.1. Probiotic–Prebiotic Synergy in Poultry Nutrition

Synbiotics are specific combinations of probiotics and prebiotics designed to enhance probiotic survival, colonization, and function in the digestive system. This synergy increases microbial balance by selectively stimulating probiotic implantation [104]. Prebiotics support this by providing substrates for probiotic survival and metabolism within the host gastrointestinal tract, enabling them to thrive despite the harsh environment [105]. This synergism enhances colonization stability and the efficiency of beneficial microbial populations, which are crucial for gut homeostasis in poultry [103].

Synbiotics are gaining increased recognition in poultry nutrition for promoting growth without the need for antibiotics. They modulate gut microbiota, thereby improving nutrient digestion and absorption in poultry [106]. Studies have showed that they enhance digestion, growth performance, and feed conversion efficiency in broilers. Rather than simply contributing to the already abundant SCFA pool, these synbiotics drive targeted shifts in the production profiles of these specific beneficial metabolites, which in turn support gut integrity and immune function [104,107]. This integrated approach offers a functional and sustainable strategy for optimizing poultry health and productivity in antibiotic-free production systems [105].

### 5.2. Effects of Synbiotics on Gut Health, Immunity, and Productivity

The use of synbiotics has been found to greatly improve the gut health by enhancing the diversity of microorganisms and stabilizing intestinal microbiota. This is important for ensuring proper digestion in poultry [108]. They also improve intestinal morphology in terms of villus length, as well as absorption [109]. As per the recent reports, intestinal shape and growth performance in poultry were all markedly enhanced by synbiotic supplementation [110]. Furthermore, synbiotics are important for the stimulation of the innate and adaptive immune systems. This involves immune cell stimulation and cytokine regulation, thus boosting resistance to disease among poultry [111]. They also strengthen the gut–liver axis to enhance metabolic and immunological performance [112].

The synergistic effect of synbiotic components promotes growth and production efficiency through improved nutrient digestibility and prevention of pathogen colonization [106]. Additionally, synbiotics increase antioxidant levels and decrease oxidative stress, which are important factors for poultry health and performance [113]. Synbiotics further contribute to environment conservation through improved nutrient utilization efficiency and a reduction in the emission of toxic metabolic products [18]. Overall, the incorporation of synbiotics into poultry feed can be viewed as an effective strategy for improving animal welfare and productivity [108].

### 5.3. Postbiotics and Paraprobiotics: Concepts and Applications in Poultry

Postbiotics and paraprobiotics can be considered alternative approaches to probiotics, providing health benefits while overcoming difficulties related to bacterial survival and stability. Postbiotics refer to metabolites, such as enzymes, peptides, and SCFAs, with positive effects on host organisms [19]. Meanwhile, paraprobiotics refer to inactivated bacteria possessing immunomodulatory and functional characteristics that make them a better choice for poultry feed applications than live bacteria due to their higher stability [114]. The use of postbiotics and paraprobiotics is especially relevant when probiotic viability is problematic [115].

Postbiotics show considerable promise for poultry applications, including improved gut health, enhanced immune response, and inhibition of pathogenic bacteria via antimicrobial metabolites [108,116]. These metabolites help to improve integrity of the intestine and microbiota balance, ultimately optimizing the performance of poultry [117]. Moreover, postbiotics and paraprobiotics offer a natural substitute for antibiotics in view of their host immune-modulatory effects and pathogen reduction, without developing resistance [62]. The suitability of these products is also enhanced by their stability and easy storage, along with their compatibility with feed manufacturing processes [108]. This increased attention towards the use of such microbial materials reflects the trend for adopting safer and more sustainable means of feeding poultry with a greater focus on functionality than viability [19].

## 6. Delivery Systems and Formulation Technologies

The efficiency of probiotic supplements depends greatly on the mode of delivery and formulation used. Several innovations such as microencapsulation, optimized delivery modes, and early-life microbiome intervention improve probiotic survivability and functionality, enhancing poultry performance [118,119,120,121]. These innovations maximize probiotic potential by improving their viability, stability, and functionality while overcoming the obstacles that limit their efficacy when administered to live birds. Table 3 provides an overview of delivery systems and formulation technologies [34,118,119,120,121,122,123,124,125,126,127,128,129,130,131].

The delivery strategies in Table 3 highlight the role of formulation technologies in maximizing probiotic efficacy in poultry systems. These approaches not only improve microbial survival and stability but also enable targeted and sustained release within the gastrointestinal tract. Among these, microencapsulation and controlled-release systems have emerged as particularly effective solutions for overcoming environmental challenges associated with probiotic application.

### 6.1. Microencapsulation and Controlled-Release Systems

Microencapsulation technology is an important method for improving probiotic efficacy in poultry farming operations. In this technology, probiotics are encapsulated using matrices to protect them against environmental factors including heat, oxygen, and gut acid [120]. This physical protection significantly increases probiotic survival rates during the processes of manufacture and digestion [121]. Materials used for microencapsulation include natural polymers such as alginate, chitosan, whey protein, maltodextrin, and complex lipid-based matrices. These diverse structural matrices offer tailored protection profiles, forming gel structures that help in the controlled release of probiotics in the intestines [123]. These systems enable targeted delivery to specific sites in the gastrointestinal tract to ensure that they colonize successfully and perform efficiently [122]. Recent developments focus on improving encapsulation methods to enhance the efficacy of various probiotics, including *Bifidobacterium* species and yeasts, in poultry production [132]. Furthermore, new microencapsulation technologies such as *Saccharomyces boulardii* encapsulated in alginate have been developed and have been shown to exhibit greater stability and improved delivery characteristics [133]. In addition, microencapsulation improves performance and gut health through sustained delivery of the probiotics [134].

### 6.2. Probiotic Administration

The probiotic administration method determines their effectiveness in poultry production. Probiotics can be added to either feed or drinking water, each of which presents its own strengths and weaknesses. Probiotics may be easily administered via feed since they are easy to administer to a flock of chickens and can be distributed evenly [118]. This ensures that the animals receive adequate doses of the probiotics, leading to improved gut health and productivity [34]. Nevertheless, the probiotics may not survive well in high temperatures, which may be experienced during the preparation of the feed [124]. On the other hand, probiotic administration through water administration provides more flexibility and rapid absorption, especially under stressful conditions or outbreaks of diseases. Water administration of probiotics is absorbed rapidly by birds and used efficiently, making it ideal for short-term treatment [125]. Recent studies have explored innovative oral administration techniques, such as pH-sensitive probiotics that offer protection to the probiotics while passing through the upper gastrointestinal tract and releasing the probiotics in the intestine [132]. It is thus necessary to choose an effective administration technique in order to maximize the efficiency of the probiotics [118].

### 6.3. Stability During Feed Processing and Storage

Maintaining probiotic stability during feed processing and storage remains a key problem in poultry nutrition, as probiotic cell viability can be greatly decreased by various environmental factors, including heat, humidity, and oxygen [121]. Processing techniques like pelleting can cause a considerable reduction in the number of live probiotic organisms, affecting their efficacy [127]. Stable probiotic formulations, especially those made of spore-forming bacteria, such as *Bacillus* spp., have been successfully developed as a means of solving the problem related to probiotic sensitivity to unfavorable conditions [126]. These formulations have the ability to sustain viability through feed processing and storage, allowing for continuous probiotic activity to the animals [121]. Besides the issue of microbial viability, another benefit of probiotic supplementation has been found to be an improvement in product quality in storage, as evidenced by increased oxidation resistance and better meat qualities [128]. In addition, probiotics also mitigate heat stress effects on meat quality and protein functionalities, indicating their usefulness in sustaining product quality in storage [129]. Improvements in probiotic formulations, such as encapsulation and coatings, are essential to increase microbial viability and product shelf-life [127].

### 6.4. In Ovo and Early-Life Microbiome Programming

In ovo or early-life interventions represent novel methods to build a beneficial gut microbiota in birds as early as possible. Such interventions include probiotics or bioactive molecule delivery, either to eggs or to newly hatched animals, to promote microbial colonization and maturation of their immunity [119]. Early-life programming stimulates intestinal development, improves nutrition intake, and boosts immune reactions in broilers. Targeting the early-life microbial colonization window achieves such goals [135]. Recent research has indicated that in ovo supplementation with probiotics can have a significant impact on gene expression associated with immune responses and metabolism, leading to increased disease resistance and productivity [136]. Also, the use of prophybiotics, a novel formulation combining probiotics and phytobiotics (plant-derived bioactive compounds) to stimulate chickens in ovo is proving effective in improving gut health and productivity levels of broilers [137]. Moreover, there is evidence that early-life nutrition programs affect long-term poultry health and productivity through their effects on the composition of gut microbiota and immune response [131]. Thus, early-life programming offers a forward-thinking approach for antibiotic-free poultry production [138].

## 7. Impact of Next-Generation Probiotics on Poultry Health and Productivity

**Figure 4:**
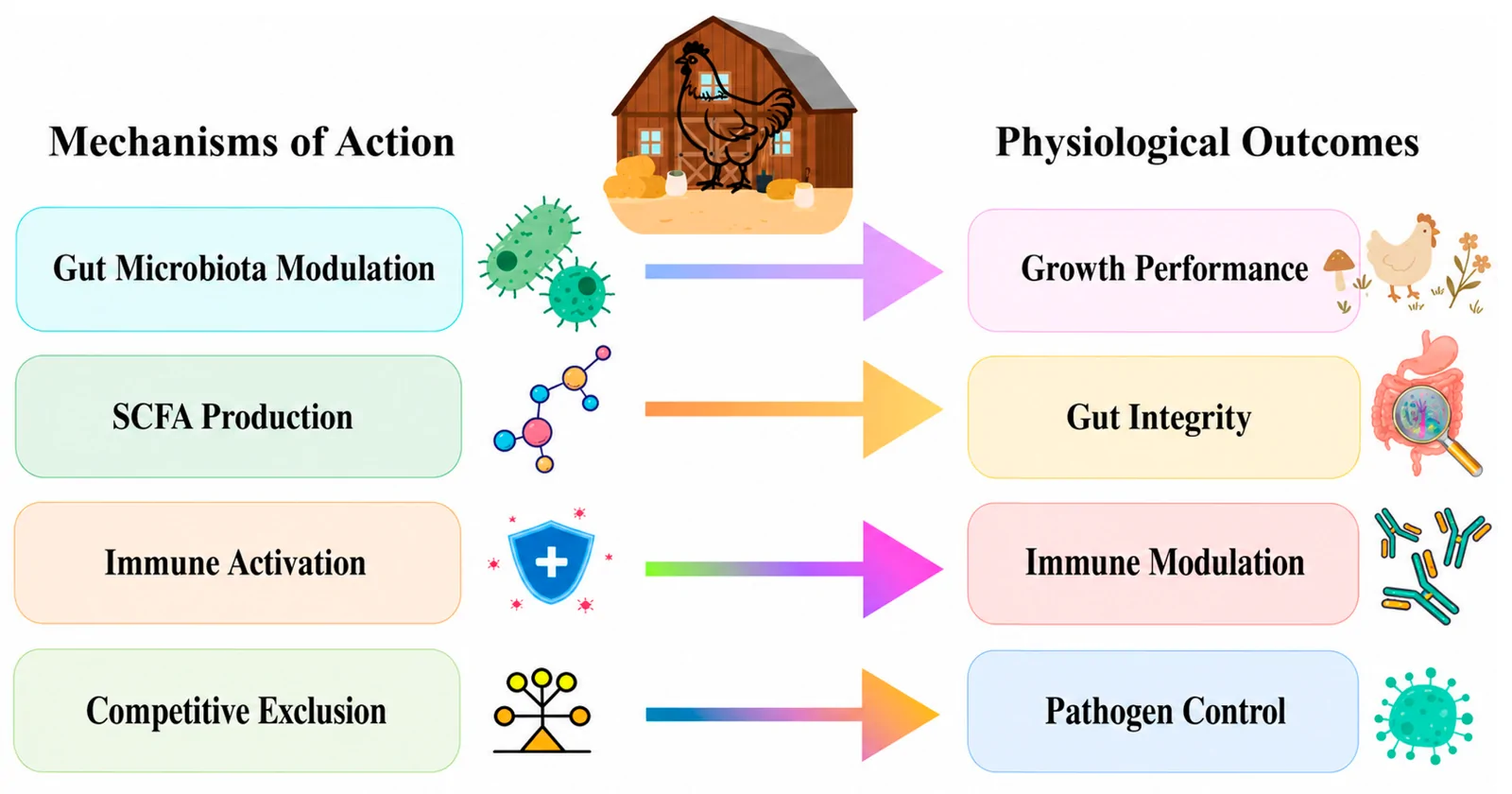
A functional representation of probiotic–prebiotic synergy from selective stimulation of beneficial microbes to enhanced colonization, SCFA production, improved gut integrity, increased productivity, and sustainable poultry production [57,142,143,144].

NGPs, now regarded as highly efficient functional feed additives for poultry, exhibit a wide array of positive impacts on both animal health and production. Unlike traditional probiotics, NGPs provide additional biological functions, such as improving digestion and immune response, as well as providing better antimicrobial activity [57,139,140,141]. Figure 4 provides an extensive overview of benefits from incorporating NGP into poultry diets [57,142,143,144].

NGPs initiate a cycle beginning with selective stimulation of gut microbiota, leading to microbial colonization and increased synthesis of metabolically active molecules like SCFAs. These metabolites enhance gut integrity, immunity, and nutrient uptake. This integrated approach improves poultry productivity and supports more sustainable production systems with reduced environmental impact.

### 7.1. Growth Performance and FCR

NGPs showed great promise in boosting growth performance and feed efficiency in chickens, due to their ability to promote optimal nutrient utilization and metabolism. These innovative probiotic strains improve gut efficiency, thereby enhancing growth and FCR [139]. Their role in improving growth performance is well-documented, based on their ability to enhance digestion and alter gut microbiome composition [140]. While traditional spore-forming probiotics such as *Bacillus subtilis* establish a baseline for enhancing growth performance, antioxidant status, and immunological responses, especially when heat stress is present [145], precision-selected NGPs are designed to achieve superior feed efficiency and targeted modifications of the cecal microbiota composition. Through such mechanisms, NGPs enhance nutrient absorption [146]. Moreover, probiotic efficacy in improving FCR depends on parameters like dose, method of administration, and strain specificity, underscoring the need for optimal approaches to probiotic use in the poultry industry [147]. In summary, incorporating NGPs into poultry feed offers a sustainable solution to increase productivity without relying on antibiotic growth promoters [139].

### 7.2. Gut Integrity and Intestinal Morphology

Gut integrity plays an essential role in poultry health and productivity. NGPs improve gut morphology, structure, and barrier function. They enhance villus height epithelial integrity, promoting effective nutrient absorption [139]. Broiler chickens have shown comparable changes in intestinal shape and gut health parameters after receiving *Lactobacillus* supplements [148]. Probiotic intake maintains gut integrity by regulating microbiome population and protecting against structural damage [149]. Improved morphological structure correlates with increased digestion efficiency and decreased incidence of enteritis disorders [150]. Additionally, NGPs influence gut health through their effects on gut microbiota, which regulate gut development and functioning [144]. These cumulative improvements result in positive growth and health outcomes for poultry [147].

### 7.3. Immune Modulation

NGPs modulate the immune system through the activation of both innate and adaptive immunity in poultry. They stimulate immune cell activity and enhance the production of key effector and regulatory cytokines, such as interferon-gamma, interleukin-6, and interleukin-10, thereby improving host immune responses against pathogen infections [57]. Effective gut microbiota immune system interactions are essential for poultry health, which support probiotics through improved gut balance and immunomodulation [149]. NGPs improve bird immunity, reducing the risk of diseases among other health disorders in stressful situations [151]. Additionally, recent studies have suggested that microbiomes can be used in boosting vaccine response and next-generation immunological interventions in poultry [152]. These findings underscore the importance of NGPs as functional agents for improving immune competence and disease resistance in modern poultry production systems.

### 7.4. Control of Enteric Pathogens

Enteric pathogen control is a major challenge in poultry production, and NGPs offer an efficient solution for managing their colonization and infection. NGPs prevent gastrointestinal bacterial infection through multiple modes of action, including competitive exclusion, antimicrobial production, and intestinal microbiome modification [141]. Various experiments have indicated that the administration of probiotics and synbiotics helps to lower colonization of *Salmonella typhimurium* and *Clostridium perfringens* [142]. Moreover, the effectiveness of pathogen suppression can also be increased through the capability of probiotics to create inhibitors as well as to compete with other microorganisms for nutrition and attachment [153]. The application of probiotics can also lead to better performance among poultry that have acquired infections from enteric pathogens, showing its possible treatment value [154]. Knowledge of the pathogenesis of pathogens like *C. perfringens* can also help in the formulation of effective probiotic treatments [155].

### 7.5. Potential Role in Reducing AMR

The rising AMR has prompted the search for new solutions in poultry farming, positioning NGPs as a potential alternative. Probiotics play an important role in lowering antibiotic dependency because they promote natural disease immunity and gastrointestinal health [156]. NGPs are helpful in combating AMR by preventing the growth of harmful bacteria, thus eliminating selective pressures created by antibiotics [143]. Moreover, probiotics regulate the gut resistome, influencing the prevalence and expression of resistance genes in microbes [157]. Nevertheless, there are still issues related to the transfer of resistance genes from probiotics to pathogenic bacteria, making it necessary to select and evaluate suitable probiotic strains [158]. Addressing these issues is essential for safely using probiotic antibiotic alternatives in the poultry industry [159].

## 8. Challenges and Knowledge Gaps

Although NGPs are rapidly advancing, critical evaluation of the available literature reveals significant gaps in knowledge and practice, limiting their application in poultry systems. Strain-specific variability and host–microbe interaction are major challenges, as NGP efficacy depends heavily on microbial genetics, host physiology, and environmental conditions, leading to inconsistent outcomes across different production settings [56,66]. Moreover, the idea of personalized or precision probiotics is the most promising solution and is at a very primitive phase in understanding the dynamics and functional responses between the host and the microbiome [59,68].

The other significant gap is reproducibility and field-level validation, where most studies illustrating the effectiveness of NGPs have been under controlled experimental conditions, which is unrealistic for describing the commercial poultry environment [57]. The differences in farm management processes, environmental marginalizing factors, and diet content tend to result in inconsistent performance, which supports the necessity for large-scope and multi-location validation studies [160]. Although there have been rapid multi-omics technologies, limited controllable incorporation of these data into practice exists, which limits the prospects of transforming the research results into practical solutions in the field solution [55,63].

One of the most significant issues is the regulatory approval processes for NGPs in particular and engineered probiotic strains specifically, since the existing structures are convoluted, taxing, and not harmonized worldwide [58]. Stringent safety regulations, such as measuring genomic stability, assessing the lack of virulence factors, and examining the potential risk of antimicrobial resistance, are very time-consuming for commercialization [71,99]. These concerns are particularly important from a One Health perspective, where antimicrobial resistance remains a shared challenge across animal, human, and environmental health sectors [161]. These regulatory ambiguities represent innovation barriers that restrict the mass implementation of advanced probiotic technologies [54]. Additionally, consumer perception of engineered microbes represents a crucial socio-economic inhibitor due to widespread skepticism about genetically engineered or bioengineered probiotics that dominate the market, regardless of their potential advantages [59]. Concerns about food safety, ethical considerations, and environmental issues further hinder their adoption, highlighting the need for better communication and public engagement [99].

In addition, significant gaps remain regarding the long-term ecological and functional effects of NGPs, particularly concerning their survival in the gut microbiome and their possible role in antimicrobial resistance processes [158,159]. The threat of HGT and its effects on the gut resistome cannot be thoroughly evaluated to ensure accessibility biosafety and sustainability [158,162]. Furthermore, translating promising laboratory findings into economically viable commercial applications remains challenging, emphasizing the need for cost-effectiveness assessments and large-scale production studies [163]. Taken together, all these issues suggest the need for an integrated and interdisciplinary method of research, including microbiology, genomics, and systems biology, to learn more about NGP functionality and improve their usage [63,68]. Consistent methodologies, robust field validation, regulatory crystallization, and enhanced consumer awareness will be crucial to advance NGPs, enabling the transition of this innovative and powerful concept into practical tools for sustainable and poultry production [54,58].

## 9. Future Perspectives

Integrating biotechnology, data science, and sustainable farming will unlock NGPs’ potential in poultry production. Accurate microbiome engineering makes it possible to design host-specific probiotics based on genetic, physiological, and microbiome profiles of birds, which enhances the stability and efficacy of colonization. Multi-omics approaches (genomics, transcriptomics, proteomics, and metabolomics) identify and characterize novel microbial strains with specific properties such as pathogen inhibition, immune modulation, or efficient nutrient metabolism. Additionally, artificial intelligence and machine learning can be used to analyze intricate biological data to forecast strain performance, maximize the combinations of microbes, and enhance delivery methods, thereby lessening the time involved in experimental research and boosting formulation effectiveness. Furthermore, translating promising laboratory findings into economically viable commercial applications remains challenging, emphasizing the need for cost-effectiveness assessments and large-scale production studies [163]. Implementing NGPs in poultry production systems is a viable solution that offers a sustainable solution to the problem of dependence on antibiotics, increased feed efficiency, and reduced effects on the environment. They enhance animal health, productivity, and resilience, aligning with global objectives for sustainable and antibiotic-free food production. Further advancement requires interdisciplinary cooperation, strong field validation, harmonization of regulations, and stakeholder awareness.

## 10. Conclusions

NGPs represent a transformative advancement in poultry nutrition, offering a promising alternative to AGPs amid rising AMR and growing demand for sustainable production systems. This review highlights how NGPs, supported by advances in microbiome science, multi-omics technologies, and synthetic biology, provide enhanced functionality through improved gut colonization, targeted pathogen inhibition, and precise host immune modulation. Compared to traditional probiotics, NGPs offer superior potential for optimizing growth performance, feed efficiency, gut integrity, and poultry health. Furthermore, emerging strategies including engineered probiotics, synbiotics, and advanced delivery systems have expanded microbial interventions capabilities, enabling more effective, stable applications in commercial conditions. However, challenges including strain specificity, regulatory complexities, large-scale validation, and consumer acceptance still limit widespread adoption. Looking ahead, the integration of precision microbiome engineering, artificial intelligence, and sustainable farming will drive future innovation in this field. Ultimately, NGPs offer significant potential to reshape modern poultry production through improved productivity, enhanced animal health, and contributions to global food security via environmentally responsible practices.

## Figures and Tables

**Table 1:** Conventional probiotics in poultry: strains, sources, mechanisms, and benefits.

Probiotic Strain/Species	Source	Poultry Type	Reported Benefits	Mechanism of Action	Reference
*Lactobacillus acidophilus*	Gut microbiota	Broilers	Improved feed conservation ratio (FCR), growth performance, gut stability	Competitive exclusion, lactic acid production, pH reduction	[2,28,29]
*Lactiplantibacillus plantarum*	Fermented products	Broilers	Reduced pathogen colonization, improved gut health	Bacteriocin production, antimicrobial activity	[5,30,31]
*Bacillus subtilis*	Soil/commercial probiotic	Broilers	Enhanced digestion, improved weight gain, feed efficiency	Enzyme secretion (protease, amylase), spore stability	[32,33,34]
*Bifidobacterium bifidum*	Intestinal microbiota	Layers	Improved immunity and gut microbiota balance	Short-chain fatty acids (SCFAs) production, microbial modulation	[12,14,35]
*Saccharomyces cerevisiae*	Yeast	Broilers	Improved nutrient utilization, immune response, overall performance	Immune stimulation, enzyme activity, gut microbiota support	[27,36,37]

**Table 2:** Engineering strategies, functional applications, and challenges of engineered probiotics in poultry systems.

Category	Strategy/Tool	Functional Application in Poultry	Key Mechanism	Reference
Antimicrobial Strategy	AMP production	Targeted pathogen inhibition, reduced antibiotic use	Heterologous expression of AMPs, in situ antimicrobial production	[78,79]
Pathogen Targeting	Receptor-mediated binding and sensing systems	Specific pathogen control, reduced off-target effects	Signal detection and targeted antimicrobial response	[8,10]
Vaccine Delivery	Antigen-expressing engineered probiotics	Enhanced mucosal immunity, disease prevention	Expression of antigens, cytokines, immune modulators	[80,81]
Nutrient Utilization	Enzyme-secreting probiotics (phytases, proteases)	Improved digestion, feed efficiency, growth performance	Enzymatic breakdown of complex feed components	[26,82]
CRISPR Technology	CRISPR-Cas gene editing	Precision modification of probiotic strains	Targeted gene insertion/deletion, metabolic control	[72,75]
Synthetic Biology	Genetic circuits and smart probiotics	Responsive pathogen detection and control	Signal sensing and conditional gene expression	[83]
Biohybrid Systems	Engineered delivery platforms	Targeted delivery of bioactive compounds	Controlled release within gastrointestinal tract	[77]
Stability Enhancement	Lyophilization, cryoprotectants	Improved shelf-life and field applicability	Preservation of viability and functionality	[84]
Biosafety & Regulation	Risk assessment frameworks	Safe commercialization and application	Evaluation of AMR transfer, toxicity, ecological impact	[70,85]

**Table 3:** Delivery systems and formulation strategies for probiotics in poultry.

Delivery Strategy	Key Features	Functional Benefits	Reference
Microencapsulation and Controlled Release	Encapsulation using polymers (e.g., alginate) for targeted intestinal delivery	Improved viability, protection from stress, sustained release, enhanced gut colonization	[120,122,123]
Feed-based Delivery	Incorporation into feed during formulation	Uniform intake, improved growth performance	[34,124]
Water-based Delivery	Administration via drinking water	Rapid delivery, suitable for stress/disease conditions	[118,125]
Stability-enhanced Formulations	Use of spore-forming strains and protective coatings	Improved thermal stability, shelf-life, and storage viability	[121,126,127]
Processing & Storage Optimization	Techniques to reduce heat/oxidative damage	Maintains probiotic activity, improves product quality	[127,128,129]
In ovo and Early-life Programming	Probiotic administration during embryonic/early stages	Early gut colonization, improved immunity and long-term performance	[119,130,131]

## Data Availability

No new data were created or analyzed in this study. Data sharing is not applicable to this article.

## Abbreviations

AGPs	Antibiotic Growth Promoters
NGPs	Next-Generation Probiotics
AMR	Antimicrobial Resistance
SCFA	Short-Chain Fatty Acid
CRISPR	Clustered Regularly Interspaced Short Palindromic Reports
FCR	Feed Conversion Ratio
GMOs	Genetically Modified Organisms
HGT	Horizontal Gene Transfer
